# Interaction of PTPIP51 with Tubulin, CGI-99 and Nuf2 During Cell Cycle Progression

**DOI:** 10.3390/biom2010122

**Published:** 2012-02-23

**Authors:** Alexander Brobeil, Michaela Graf, Moritz Eiber, Monika Wimmer

**Affiliations:** Institute of Anatomy and Cell Biology, Justus-Liebig-University, 35392 Giessen, Germany; Email: michaela.graf@anatomie.med.uni-giessen.de (M.G.); moritz.eiber@med.uni-giessen.de (M.E.); monika.wimmer@anatomie.med.uni-giessen.de (M.W.)

**Keywords:** PTPIP51, FAM82A2, FAM82C, RMD-3, mitosis, cell cycle, microtubule, CGI-99, Nuf-2, EGFR

## Abstract

Protein tyrosine phosphatase interacting protein 51 (PTPIP51), also known as regulator of microtubule dynamics protein 3, was identified as an *in vitro* and *in vivo* interaction partner of CGI-99 and Nuf-2. PTPIP51 mRNA is expressed in all stages of the cell cycle; it is highly expressed six hours post-nocodazole treatment and minimally expressed one hour post-nocodazole treatment. Recent investigations located PTPIP51 protein at the equatorial plate. This study reports the localization of the PTPIP51/CGI-99 and the PTPIP51/Nuf-2 complex at the equatorial region during mitosis. Moreover, Duolink proximity ligation assays revealed an association of PTPIP51 with the microtubular cytoskeleton and the spindle apparatus. High amounts of phosphorylated PTPIP51 associated with the spindle poles was seen by confocal microscopy. In parallel a strong interaction of PTPIP51 with the epidermal growth factor receptor phosphorylating PTPIP51 at the tyrosine 176 residue was seen. In the M/G1 transition a high level of interaction between PTPIP51 and PTP1B was registered, thus restoring the interaction of PTPIP51 and Raf-1, depleted in mitotic cells. Summarizing these new facts, we conclude that PTPIP51 is necessary for normal mitotic processes, impacting on chromosomal division and control of the MAPK pathway activity.

## 1. Introduction

Protein tyrosine phosphatase interacting protein 51 (PTPIP51) is a multifunctional protein with implication in processes like proliferation, differentiation and apoptosis [1,2]. To fulfill these opposite functions the PTPIP51 protein possesses different domains necessary for protein-protein interaction and subcellular localization [1,3,4].

Data for the involvement of PTPIP51 in mitogenic processes are limited. Mitosis is characterized by four stages, named prophase, anaphase, metaphase and telophase, controlled by various checkpoints [5]. Moreover, distinct signaling pathways are required for entering mitosis. The mitogen activated protein kinase pathway was identified to play a critical role in the G2/M transition [6]. During mitosis itself, Raf-1 is tightly regulated by the raf kinase inhibitory protein (RKIP), which titrates the signal of the MAPK pathway ensuring normal chromosomal segregation. RKIP-depleted cells display chromosomal abnormalities due to incorrect spindle assembly [7]. PTPIP51 is linked to the MAP pathway on the level of Raf-1 [3]. As described recently, these interactions are regulated by the tyrosine phosphorylation status of PTPIP51. Hyperphosphorylation of PTPIP51, as induced by pervanadate inhibits the interaction with 14-3-3β and Raf-1, respectively [8]. Highly tyrosine phosphorylated PTPIP51 also was observed in blasts of acute myeloid leukemia (AML). In addition, due to the lack of the phosphatase PTP1B, PTPIP51 cannot be dephophorylated in cells of AML [9]. These leukemic cells feature autonomous proliferation with a high mitotic index [10]. Thus, phosphorylated PTPIP51 might be involved in proliferation processes of normal as well as cancer cells. The phosphorylation of PTPIP51 might be regulated by the presence or mutation of its interaction partners.

PTPIP51 is alternatively named regulator of microtubule dynamics protein 3 (RMD-3). This protein family of microtubule associated proteins comprises three members, named RMD-1, RMD-2 and RMD-3 [1]. RMD-1 was identified to play a key role in mitogenic processes, especially in the correct segregation of the chromosomes during mitosis [11]. Notably, in mitotic cells PTPIP51 is associated with the mitotic spindle apparatus. Error-free chromosome segregation is only achieved if PTPIP51 provides stable attachment of the spindle apparatus to the kinetochores [11]. Yet, the exact mechanism of spindle assembly to the kinetochores remains elusive [12]. As has been revealed by co-immunoprecipitation experiments, PTPIP51 interacts with two mitotic proteins, namely CGI-99 and Nuf-2 [1,2].

The human Nuf2 (hNuf2) protein is associated with the outer kinetochore plate and is essential for correct spindle attachment to the kinetochore plate [13]. In addition, the centrosome is also essential for the formation of the mitotic spindle apparatus. Human ninein (hNinein) is one component of the pericentriolar matrix stabilizing the linkage between the microtubules and the centrioles [14]. CGI-99 is an interaction partner of hNinein, a protein associated with the centrosome. Of note, CGI-99 could inhibit the phosphorylation of hNinein mediated by glycogen synthase kinase 3 β, thus influencing the nucleation of the microtubules [15].

These findings suggest PTPIP51 may play a pivotal role in mitotic processes, both in chromosomal segregation and cell signal titration. Therefore, we investigated the functional implication of PTPIP51 in cell cycle progression in a human keratinocyte cell line during a synchronized cell cycle. The association of microtubule formation was investigated by double-immunostainings and duolink proximity ligation assays. To gain further insights in phosphorylation events, samples were probed with a peptide specific antibody raised against the tyrosine residue 176 of PTPIP51. PTPIP51 interaction with the epidermal growth factor receptor and c-Src, the phosphatase PTP1B and Raf-1 was investigated by doulink proximity ligation assays. The acquired data suggest an involvement of PTPIP51 in correct chromosome segregation by the spindle apparatus, as well as for uncoupling Raf-1 from the MAPK pathway ensuring correct chromosomal segregation during cell cycle progression.

## 2. Experimental Section

### 2.1 Cell Culture

For all experiments of this study HaCaT cells were used kindly provided by Dr. Teschemacher (Department of Pharmacology, Justus Liebig University, Giessen, Germany) with the permission of Dr. Fusenig (DKFZ, Heidelberg, Germany, MTA number L-4598). Cells were kept at 37 °C in humidified 5% CO_2_ atmosphere and were cultured in RPMI 1640 medium (PAA, Paching, Austria, Cat.# E15-840) supplemented with 10% fetal calf serum (FCS), 100 U/mL penicillin, 100 μg streptomycin. To arrest cells in prophase they were treated with 0.1 μg/mL nocodazole for 12 hours.

### 2.2. Quantitative Reverse Transcriptase Polymerase Chain Reaction (qRT-PCR)

The total RNA from nocodazol treated cells was prepared using RNeasy kit (Qiagen) according to the manufacturer’s protocol. The isolated mRNA was reverse-transcribed using M-MuLV reverse transcriptase (Fermentas) and poly-dT primer according to standard protocol. The subsequent PCRs were done using Tag-DNA-polymerase (Fermentas). The reactions were optimized for concentration of primers and number of cycles to achieve an almost equal signal from PTPIP51 and actin (for normalization) band in the control samples (untreated cells). The best results were obtained using 22 pmol PTPIP51 and 8 pmol actin primer per 100 µL reaction mix and using 32 to 37 cycles (denaturation: 94 °C 30 sec; annealing: 58 °C 30 sec; elongation 72 °C 1 min 30 sec). The PCR products were separated on 1% agarose gel and stained with ethidium-bromide. The intensities PTPIP51 and actin PCR product were quantified with AIDA software.

### 2.3. The PTPIP51 Antibody

The PTPIP51 antibody (P51ab) was produced as described below and is consecutively named as P51ab.

The cDNA sequence encoding amino acids (aa) 131–470 was inserted into the BamHI and HindIII sites of the plasmid pQE30 and expressed as a His6-tagged protein in the protease-deficient *Escherichia coli* expression strain AD202 [araD139DE(argFlac) 169 ompT1000:kan flhD5301 fruA25 relA1 rps150(strR) rbsR22 deoC1]. The protein was purified to electrophoretic homogeneity by chromatography on an Ni-agarose column [16]. Immunization of rabbits was performed with 0.5 mg of the purified protein in 0.5 mL RIBI adjuvant, followed by booster injections with 0.5 and 0.3 mg on days 14 and 21, respectively. The antiserum was collected on day 28. Monospecific antibodies were prepared following the method described by Olmsted [17]. Briefly, 2 mg of purified antigen was blotted on nitrocellulose after SDS electrophoresis. The protein band was marked with Ponceau solution and cut out. After blocking of the membrane strip with 1% low-fat milk powder in phosphate-buffered saline, the membrane was incubated with the antiserum for 1 hour, followed by extensive washing with Tris-EDTA-buffered saline. The antibodies were eluted with 0.2 M glycine (pH 2.0) for 2 minutes, followed by immediate neutralization with 1 M triethanolamine.

The specificity of the PTPIP51 antibody was tested by ELISA and by immunoblotting of the isolated purified recombinant protein staining bands with 52 kDa, 34 kDa, and 30 kDa. Immunoblotting of homogenates from porcine spleen tissue revealed bands of 48 kDa, 40 kDa, and 29 kDa [18]. The antibody binds to the EGFP fusion PTPIP51 protein expressed in HEK293 [19]. Preabsorbing the PTPIP51 antibody against its antigen completely abolished the immune reaction in all tested samples [20,21,22].

### 2.4. Peptide Specific Phospho-Tyrosine 176 PTPIP51 Antibody

For analysis of the tyrosine phosphorylation state of PTPIP51, an antibody (BioLux, Stuttgart, Germany) to the tyrosine 176 phosphorylated sequence DAESEGGYTTANAE was used (P51ab-PTyr). Identity and purity of the synthetized peptide was approved by ESI-MS and UV-analysis. Guinea pigs were immunized with the KLH-conjugated peptides. The specificity of each antibody was tested by ELISA and Western blot. To verify the use of these peptide specific antibodies for immunostaining, preabsorption experiments were performed.

### 2.5. Preabsorption Experiments for Immunoblotting

**Figure 1:**
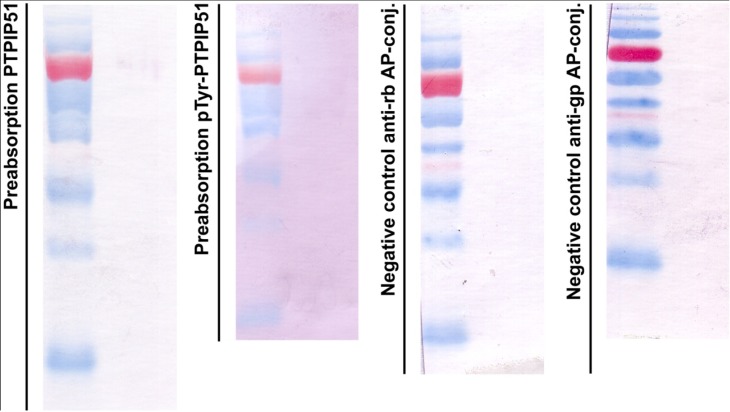
Control experiments for immunoblotting. First panel: immunoblot done with the preabsorbed P51ab-antibody. Second panel: immunoblot done with preabsorbed P51ab-PTyr antibody. Third panel: Negative control with the omission of the P51ab antibody. Fourth panel: Negative control with the omission of the P51ab-PTyr antibody.

Specificity of the PTPIP51 immunoreactivity for both antibodies P51ab and P51ab-PTyr was controlled by preabsorbing both antibodies with the corresponding purified antigen (P51ab: recombinant PTPIP51 full length protein; P51ab-PTyr: phophorylated antigenic peptide described in Section 2.4) at a concentration of 20 µg/mL for 18 hours at 4 °C prior to the immunostaining. As positive control, a normal incubation mixture including the same concentration of PTPIP51 antibody was used. Figure 1 displays in the left panel an immunoblot done with the preabsorbed P51ab-antibody. In Figure 1 right panel an immunoblot done with preabsorbed P51ab-PTyr antibody is shown.

### 2.6. Immunoblotting

Samples of HaCat cell lysate were separated on a 10% SDS-PAGE gel. Transfer on an Immobilon P membrane (Millipore) was performed according to Towbin *et al.* [23]. The membrane was blocked with 10% fat-free milk powder in PBS. Incubation with polyclonal rabbit anti-PTPIP51 (P51ab) or polyclonal guinea pig anti-pTyr176-PTPIP51 (P51ab-PTyr) was done overnight at room temperature. Either alkaline phosphatase-conjugated anti-rabbit or alkaline phosphatase-conjugated anti-guinea pig immunoglobulins were applied for 1 h at room temperature diluted in 0.5% fat-free milk powder. The reaction was visualized with the SigmaFast BCIP/NBT substrate. A prestained molecular weight marker (Biorad, Cat# 161-0374) was used for calibration.

### 2.7. Fluorescence Microscopy

The Axioplan 2 fluorescence microscope equipped with Plan-Apochromat objectives (Carl Zeiss, Jena, Germany) was used for photo documentation. For visualization of the secondary antibody Cy3-donkey-anti-rabbit an excitation filter with a spectrum of 530–560 nm and an emission filter with a spectrum 572–647 nm were used. Alexa Fluor 488 goat anti-mouse IgG was visualized by an excitation filter with a range of 460–500 nm and an emission filter with a range of 512–542 nm.

### 2.8. Confocal Laser Scanning Microscopy

Confocal images of cells were obtained with a Leica confocal laser scanning microscope (CLSM, TCS SP2, Leica, Bensheim, Germany). Confocal images of Cy3 fluorescence were acquired using Plan-Apochromat ×63/1.4 oil objective, 548 nm excitation wavelengths (helium-neon laser) and a 560–585 nm bandpass filter. The pinhole diameter was set to yield optical sections of 1 Airy unit. For the detection of Alexa 488, we used a Plan-Apochromat ×63/1.4 oil objective, the 488 nm excitation wavelength of an argon laser, and a 505–530 nm band-pass filter. The pinhole diameter was set to yield optical sections of 1 Airy unit. Confocal images of To-Pro-3 (Molecular probes, Cat.# T3605) (nuclear staining) fluorescence were acquired using Plan-Apochromat × 63/1.4 oil objective, 633 nm excitation wavelengths (helium-neon laser), the 650–670 nm bandpass filter. The pinhole diameter was set to yield optical sections of 1 Airy unit. Acquired DIC and confocal images were analyzed and combined using the LCS software (Leica Confocal Software).

Acquired images were subsequently processed by ImageJ (v1.43m; Rasband, W.S., ImageJ, U.S. National Institutes of Health, Bethesda, Maryland, USA, 1997–2011) [24] using an iterative deconvolution plug-in by Bob Dougherty [25]. Options were set for all confocal acquired images as follows: 8 numbers of iteration and 2.0 pixels of LP filter diameter. Point spread function was calculated for each channel separately by the ImageJ plug-in created by Bob Dougherty [26].

### 2.9. Intensity Correlation Analysis

Intensity correlation analysis (ICA) was carried out using ImageJ (v1.43m; Rasband, W.S., ImageJ, U.S. National Institutes of Health, Bethesda, Maryland, USA, 1997–2011) [24] and an appropriate plug-in for ICA included in the plug-in package of the Wright cell imaging facility [27,28].

### 2.10. Duolink II Proximity Ligation Assay (DPLA)

*In situ* interactions were detected by the proximity ligation assay kit Duolink II (Olink Bioscience, Uppsala, Sweden; PLA probe anti-rabbit minus, Cat.#92005–0100; PLA probe anti-mouse plus, Cat.#92001–0100; PLA probe anti-goat plus, Cat.#92003–0100; Detection Kit Orange, Cat.#92007–0100). The DPLA probe anti-rabbit minus binds to the PTPIP51 antibody, whereas the PLA probe anti-mouse plus or PLA probe anti-goat plus binds to the antibody of the probable interaction partner (see Table 1), respectively. The Duolink proximity ligation assay secondary antibodies only generate a signal when the two DPLA probes have bound, which only takes place if both proteins are closer than 40 nm, indicating their interaction [29]. PFA-fixed HaCat cells were pre-incubated with blocking agent for 1 h. After washing in PBS for 10 min, primary PTPIP51 antibody (1:1000) was applied to the samples. Primary antibodies of the interacting partners (Table 1) were used for proving the interaction by co-incubation with the PTPIP51 antibody. Incubation was done overnight in a pre-heated humidity chamber. Slides were washed three times in PBS for 10 min. Duolink II PLA probes detecting rabbit, goat or mouse antibodies were diluted in the blocking agent in a concentration of 1:5 and applied to the slides followed by incubation for 1 h in a pre-heated humidity chamber at 37 °C. Unbound DPLA probes were removed by washing two times in PBS for 5 min. The samples were incubated with the ligation solution consisting of Duolink II Ligation stock (1:5) and Duolink Ligase (1:40) diluted in high purity water for 30 min at 37 °C. After ligation the Duolink Amplification and Detection stock, diluted 1:5 with addition of polymerase (1:80), was applied to the slides for 100 min. Dapi was used to identify the nuclei. After the final washing steps the slides were dried and cover slips were applied.

**Table 1:** List of antibodies used in this study.

		Immunogen		Antibody Source		Clone	Dilution		Manufacturer		
**PTPIP51** **(P51ab)**		Human recombinant PTPIP51 protein encoding amino acids (aa) 131–470		Rabbit polyclonal			1:500		Prof. HW Hofer, Biochemical Department, University Konstanz, Germany		
**PTPIP51- anti-pTyr176** **(P51ab-PTyr)**		Purified total IgG fraction KLH-peptide conjugate		Guinea pig polyclonal			1:1000		BioLux, Stuttgart, Germany		
**Ki67**		Human recombinant peptide corresponding to a 1002 bp Ki-67 cDNA fragment		Mouse monoclonal		MIB-1	1:100		Dako Cytomation Cat.# M7240		
**CGI-99**		epitope mapping at the N-terminus of CGI-99 of human origin		Goat polyclonal		N-14		1:100		Santa Cruz Biotechnology Cat.# sc-104834
**Nuf-2 (cdcA1)**		raised against amino acids 1–300 mapping at the N-terminus of CdcA1 of human origin		Mouse monoclonal		E-6		1:100		Santa Cruz Biotechnology Cat.# sc-271251
**c-Src**		Full-length recombinant c-Src of human origin		Mouse monoclonal		17AT28		1:100		Santa Cruz Biotechnology Cat.# sc-130124
**PTP1B**		Recombinant protein corresponding to aa 1–321 of human PTP1B		Mouse monoclonal		107AT531		1:200		AbnovaCat.# MAB1152
**Raf-1**		Mapping the C-terminus of Raf-1		Mouse monoclonal		E-10		1:100		Santa Cruz Biotechnology Cat# sc-7267
**EGFR**		raised against plasma membranes of A431 cells		Mouse monoclonal		2E9		1:100		Santa Cruz BiotechnologyCat# sc-57091
**MEK1**		produced by immunizing animals with full length MEK1/2 proteins		Mouse monoclonal		L38C12		1:100		Cell Signaling Technology Cat# 4694
**Erk1/2**		produced by immunizing animals with a synthetic peptide corresponding to residues near the C-terminus of rat p44 MAP kinase.		Rabbit monoclonal		137F5		1:100		Cell Signaling Technology Cat# 4695
**Actin**		epitope corresponding to amino acids 180–375 mapping at the C-terminus of Actin of human origin		Mouse monoclonal		H-196		1:50		Santa Cruz Biotechnology Cat# sc-7210
**Vimentin**		specific for an epitope mapping between amino acids 411–447 near the C-terminus of Vimentin of human origin		Mouse monoclonal		E-5		1:50		Santa Cruz Biotechnology Cat# sc-373717
**Cy3 donkey anti-rabbit Fab fragments**				Donkey				1:1600		Dianova Cat.# 711-166-152
**Cy3 donkey anti-guinea pig**				Donkey				1:400		Dianova Cat.# N/A
**Alexa 488** **Coupled to anti-mouse antibody**		IgG heavy chains from mouse		Goat				1:800		Invitrogen Cat# A11029

**Figure 2:**
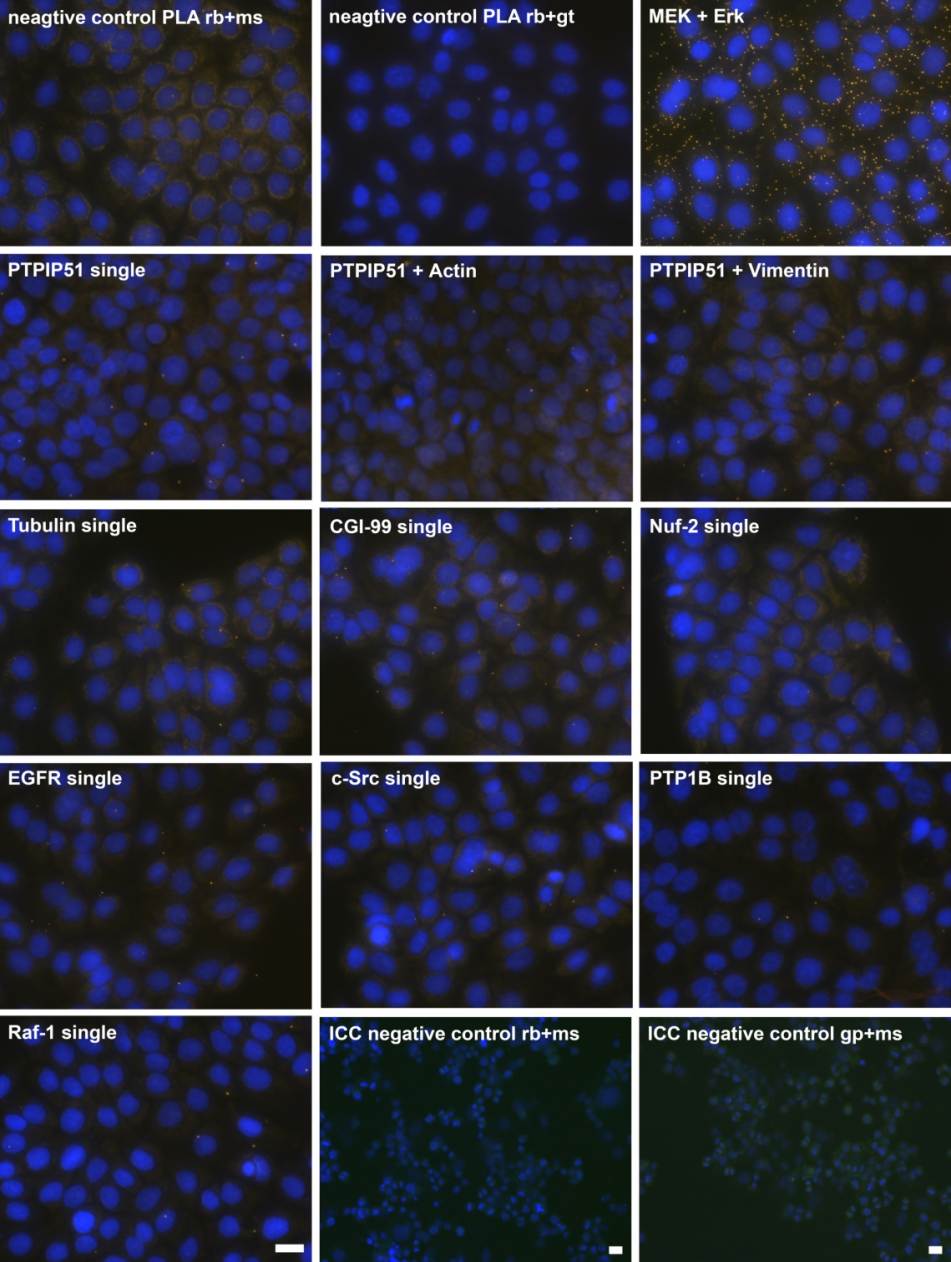
Control experiments of theDuolink II Proximity Ligation Assay (DPLA) specifity. Negative controls were done for both rabbit and mouse PLA probe and rabbit and goat PLA probe (omission of all primary antibodies). The positive control was done using two antibodies against MEK and Erk, respectively. PTPIP51 interaction with the cytoskeleton was tested by using an actin and vimentin antibody. All antibodies used for DPLA were tested in single staining experiments using the appropriate mixture of PLA probes. Immunocytochemistry (ICC) negative controls were done for the Cy3 donkey anti-rabbit/Alexa Fluor 488 goat anti mouse IgG (ICC negative control rb+ms) mixture and Cy3 donkey anti-guinea pig/Alexa Fluor 488 goat anti mouse IgG (ICC negative control gp+ms) mixture (omission of the primary antibodies for PTPIP51 and Tubulin). Bar: 20 µm.

DPLAs were controlled by performing the DPLA with either omission of both primary antibodies, omission of one primary antibody, with P51ab and an antibody detecting a non-expressed cytoplasmic protein (vimentin) or with P51ab and an antibody to a non-PTPIP51 interacting protein (actin) (Figure 2).

## 3. Results

### 3.1. Quantitative Reverse Transcriptase Polymerase Chain Reaction of Nocodazole Treated HaCat Cells

**Figure 3:**
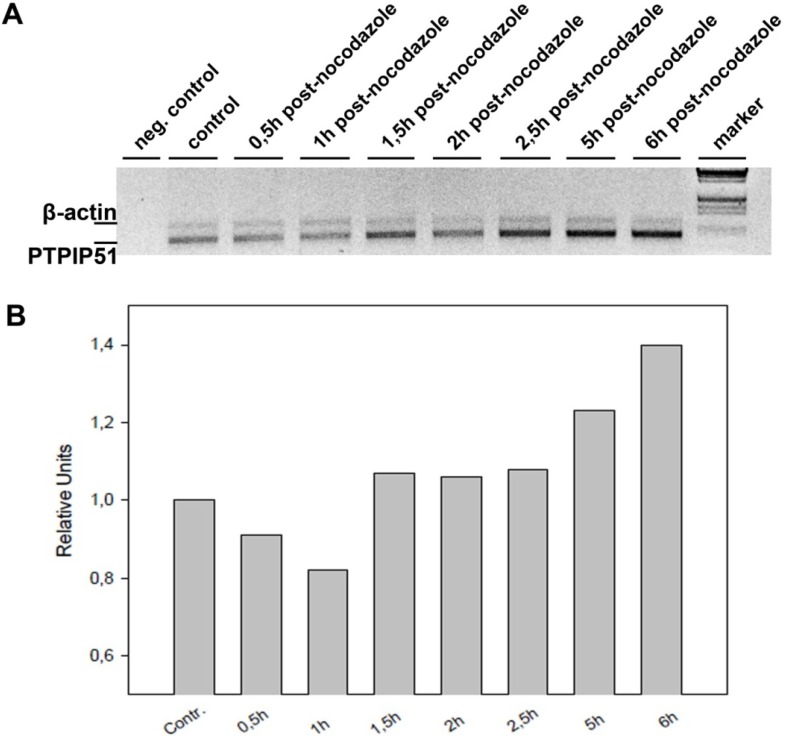
Quantitative reverse transcriptase polymerase chain reaction (qRT-PCR) of HaCat cell lysate harvested at distinct periods after nocodazole treatment. (**A**) Gel of the RT-PCR. Upper bands display β-actin and PTPIP51 mRNA is shown in the lower bands. (**B**) Relative values of PTPIP51 mRNA at different time points post-synchronization. The value for the control was set to 1. Samples were normalized to the control.

Samples of HaCat cells were analyzed at specific periods after cell cycle synchronization with nocodazole. PTPIP51 mRNA was traced in all samples. The amount of PTPIP51 is stable throughout the observation period of 2–4 hours. At 5 hours after nocodazole treatment an increase in PTPIP51 mRNA was observed. Moreover, after 6 hours post-nocodazole PTPIP51 mRNA amount was increased (Figure 3).

### 3.2. Immunoblots of HaCat Cells 2 Hours and 4 Hours after Nocodazole Treatment

Immunoblots of synchronized cells revealed bands at molecular weights of 30 kDa, 38 kDa, 45 kDa 52 kDa, 60 kDa, 65 kDa, 90 kDa, 110 kDa after 2 and 4 hours post-nocodazole treatment.

**Figure 4:**
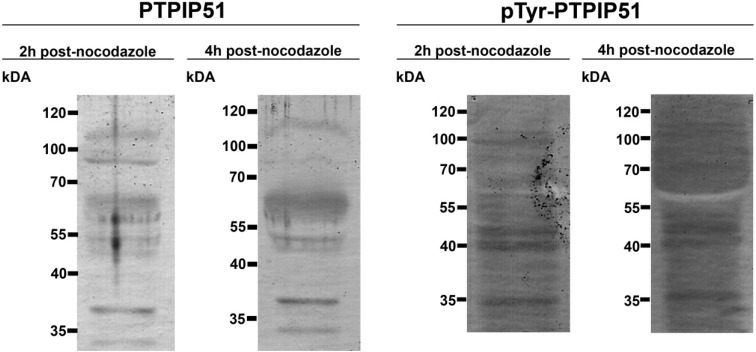
Immunoblot of nocodazole treated HaCat cells. Cell lysate was analyzed at 2 h and 4 h post-nocodazole. PTPIP51 antibody: P51ab.

Immunoblots using the peptide specific antibody against the phospho-tyrosine 176 residue of PTPIP51 revealed specific bands at molecular weights of 30 kDa, 38 kDa, 45 kDa, 52 kDa and 60 kDa, 90 kDa, 110 kDa 2 hours after restart of cell cycle. After 4 hours post-nocodazole treatment bands of 30 kDa, 37 kDa, 45 kDa, 52 kDa, 90 kDa, 110 kDa were observed. Yet, a band of 60 kDa was lacking (Figure 4).

### 3.3. PTPIP51 is Expressed in Mitotic HaCat Cells and Is Co-Localized with Tubulin During Mitosis and Interacts Directly with Tubulin

**Figure 5:**
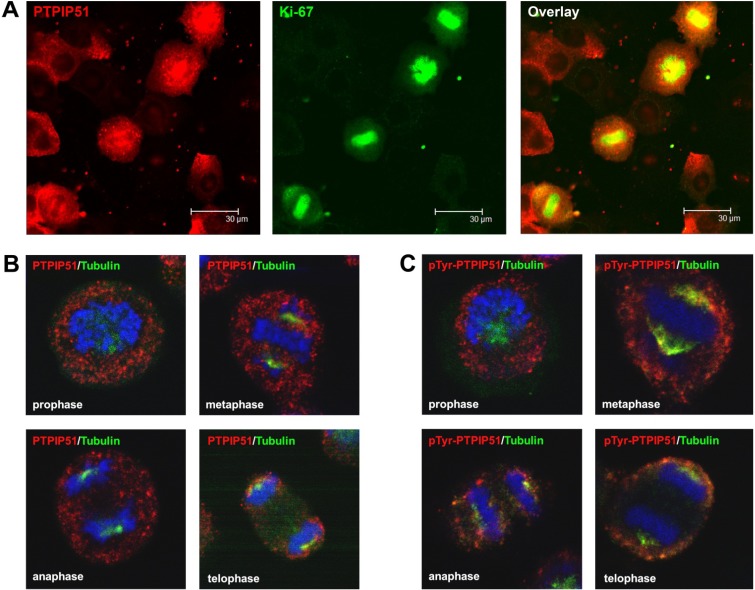
PTPIP51 protein expression during the cell cycle. (**A**) Confocal laser scanning microscopic picture of the PTPIP51 protein in proliferating HaCat cells. Cells were treated with nocodazole for cell cycle synchronization. Cells were analyzed in metaphase. PTPIP51 antibody: P51ab, Ki-67 antibody for identification of metaphase. (**B**) Confocal laser scanning microscopy of PTPIP51 and tubulin in the four mitotic stages. PTPIP51 antibody: P51ab. Nuclei marked in blue using To-Pro3. (**C**) Confocal laser scanning microscopic picture of the tyrosine 176 phosphorylated PTPIP51 protein and tubulin in the four mitotic stages. PTPIP51 antibody: P51ab-PTyr. Nuclei marked in blue using To-Pro3.

**Figure 6:**
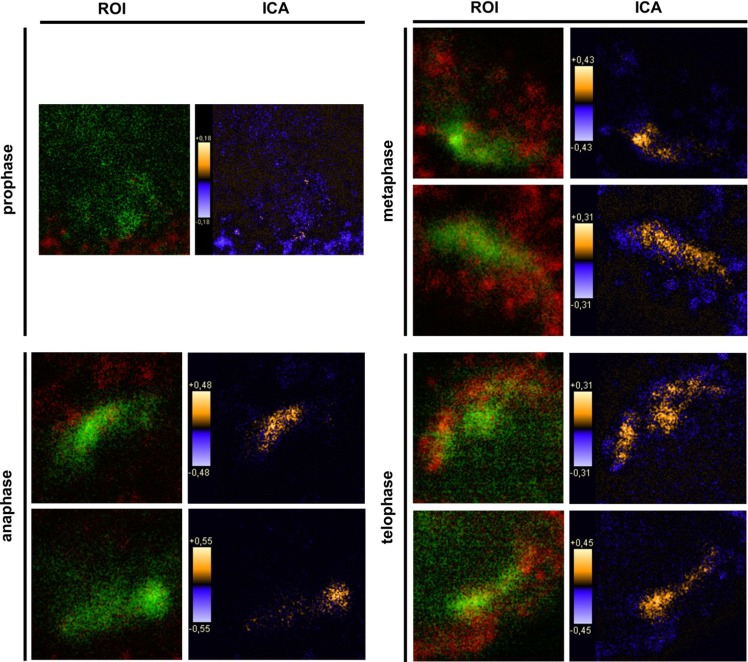
Intensity correlation analysis (ICA) of PTPIP51 and tubulin in mitotic cells. Region of interest (ROI) was set to the tubulin staining of each cell of Figure 5B. The co-localization of PTPIP51 and tubulin is displayed in orange. Sites of non-co-localization are marked in blue.

As seen in Figure 5A mitotic HaCat cells identified by Ki-67 staining displayed PTPIP51 protein, which were marked by Ki-67 staining. Investigating the four mitotic stages PTPIP51 showed a cell cycle stage dependent co-localization with tubulin. In prophase cells no co-localization for PTPIP51 and tubulin was observed (Figure 5B). In contrast, cells of the metaphase, anaphase and telophase displayed co-localization of PTPIP51 protein and tubulin. PTPIP51 overlapped with tubulin at the spindle pole (Figure 5B). The calculated co-localization by ICA, basing on the comparison of fluorescence intensities (see Materials and Methods), is displayed in Figure 6. The co-localization is indicated in yellow to orange and parts with non-co-localization are shown in blue.

**Figure 7:**
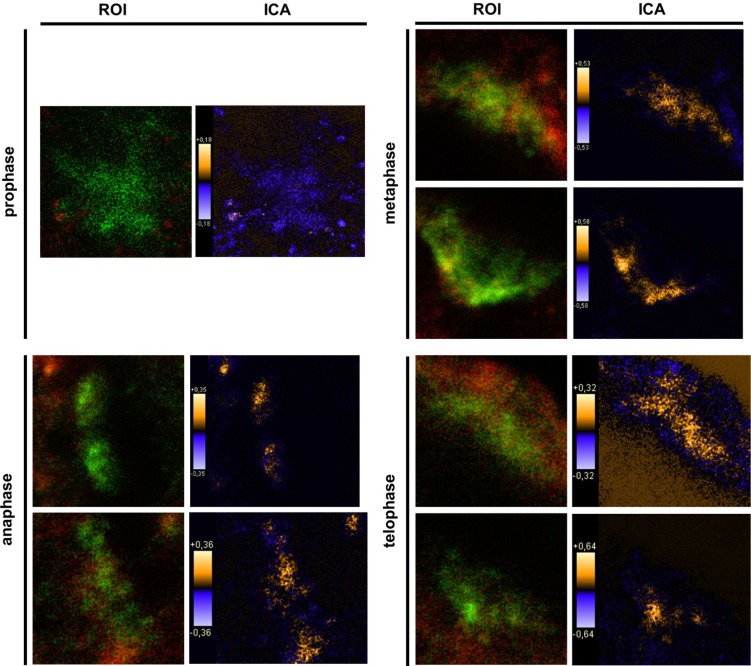
Intensity correlation analysis (ICA) of tyrosine 176 phosphorylated PTPIP51 and tubulin in mitotic cells. Region of interest (ROI) was set to the tubulin staining of each cell of Figure 5C. The co-localization of pTyr-PTPIP51 and tubulin is displayed in orange. Sites of non-co-localization are marked in blue.

Using the phospho-tyrosine specific antibody for co-localization studies revealed that PTPIP51 protein was mostly phosphorylated at its tyrosine 176 residue in all mitotic stages (Figure 5C). In prophase cells tyrosine 176 phosphorylated PTPIP51 was not co-localized with tubulin, substantiating the data seen in immunocytochemical experiments, where no co-localization of PTPIP51 and tubulin was observed. Yet, at the spindle pole tyrosine 176 phosphorylated PTPIP51 protein displayed a co-localization with tubulin corresponding to the observation in immunocytochemical experiments. The computed data of the ICA are displayed in Figure 7. Co-localization is displayed in yellow to orange and non-co-localized parts are shown in blue.

**Figure 8:**
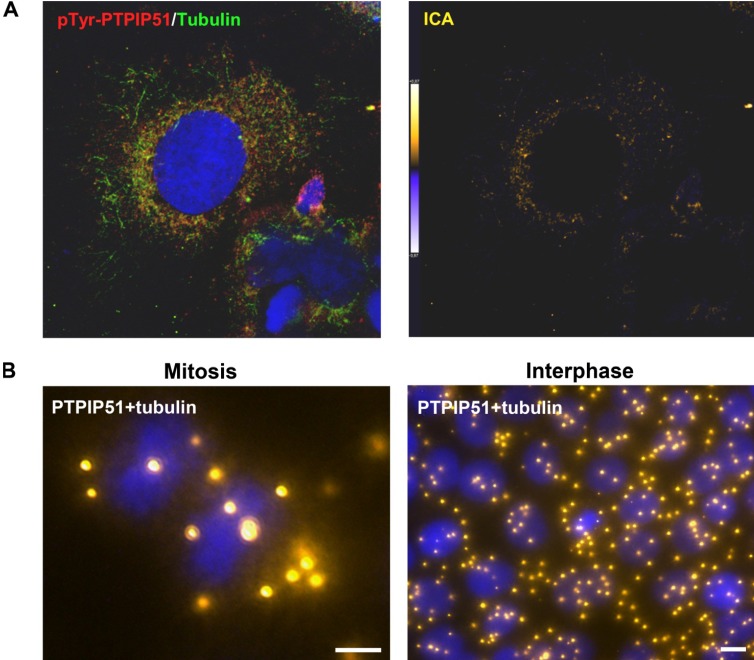
Tyrosine 176 phosphorylated PTPIP51 in HaCat interphase cells and PTPIP51/tubulin DuoLink proximity ligation assay (DPLA). (**A**) Confocal laser scanning microscopy of tyrosine 176 phosphorylated PTPIP51 and tubulin in interphase cell. PTPIP51 antibody: P51ab-PTyr. Nucleus marked in blue using To-Pro3. Intensity correlation analyses were carried out using the full image of pTyr-PTPIP51/tubulin as input. The co-localization of PTPIP51 and tubulin is displayed in orange. Sites of non-co-localization are marked in blue. (**B**) DPLA of PTPIP51 and tubulin in mitosis and DPLA of PTPIP51 and tubulin in interphase. PTPIP51 antibody: P51ab. Nuclei are marked by Dapi. Bars: 10 µm.

In interphase cells tyrosine 176 phosphorylated PTPIP51 protein also exhibited a partial co-localization with the tubulin cytoskeleton (Figure 8A). The computed data of the ICA is displayed in Figure 8A (ICA). Co-localization is displayed in yellow to orange and non-co-localized parts are shown in blue.

A direct interaction of PTPIP51 with tubulin was substantiated by the duolink proximity ligation assay in mitosis as well as in the interphase (Figure 8B). 

### 3.4. PTPIP51 Interacts with CGI-99 and Nuf-2 During Mitosis and During Interphase

**Figure 9:**
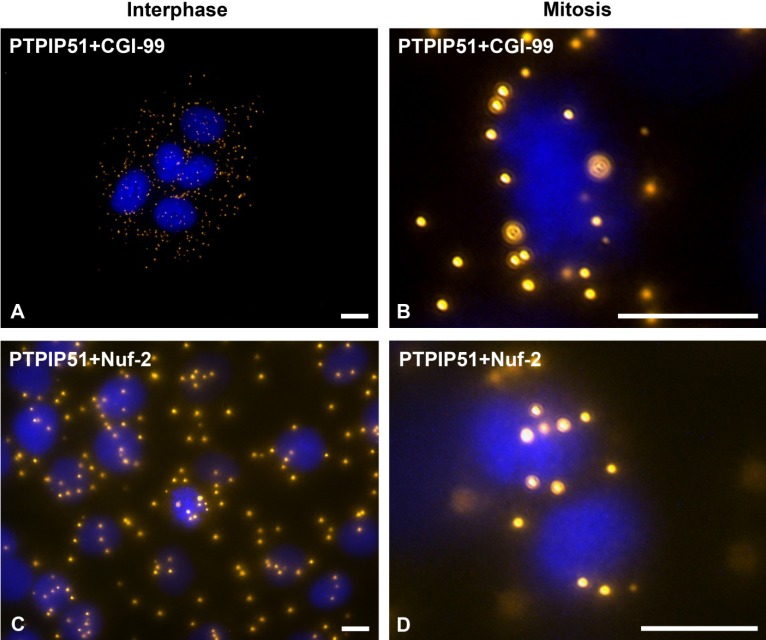
DPLA of PTPIP51 with CGI-99 and Nuf-2 in interphase and during mitosis. (**A**) DPLA of PTPIP51 and CGI-99 in interphase. (**B**) DPLA of PTPIP51 and CGI-99 in a mitotic cell of the metaphase. (**C**) DPLA of PTPIP51 and Nuf-2 during interphase. (**D**) DPLA of PTPIP51 and Nuf-2 in a mitotic cell of the metaphase. PTPIP51 antibody: P51ab. Nuclei are marked by Dapi. Bar: 10µm.

Mitotic as well as interphase cells revealed an interaction with the two mitotic proteins CGI-99 and Nuf-2 (Figure 9). In interphase PTPIP51/CGI-99 interaction was spread throughout the whole cytoplasm (Figure 9A). In mitotic cells of the metaphase PTPIP51/CGI-99 interaction was partially restricted to the region near the equatorial plate (Figure 9B). Interphase cells also displayed an interaction of PTPIP51 and Nuf-2 as seen by DPLA (Figure 9C). The interactions as indicated by the DPLA spots were dispersed throughout the whole cytoplasm and displayed a partial nuclear localization (Figure 9C). Yet, in metaphase cells the PTPIP51/Nuf-2 interaction was traced to the chromosomes of the equatorial plate (Figure 9D).

### 3.5. Interaction of PTPIP51 and the Epidermal Growth Factor Receptor, c-Src, PTP1B and Raf-1 is Cell Cycle Dependent

**Figure 10:**
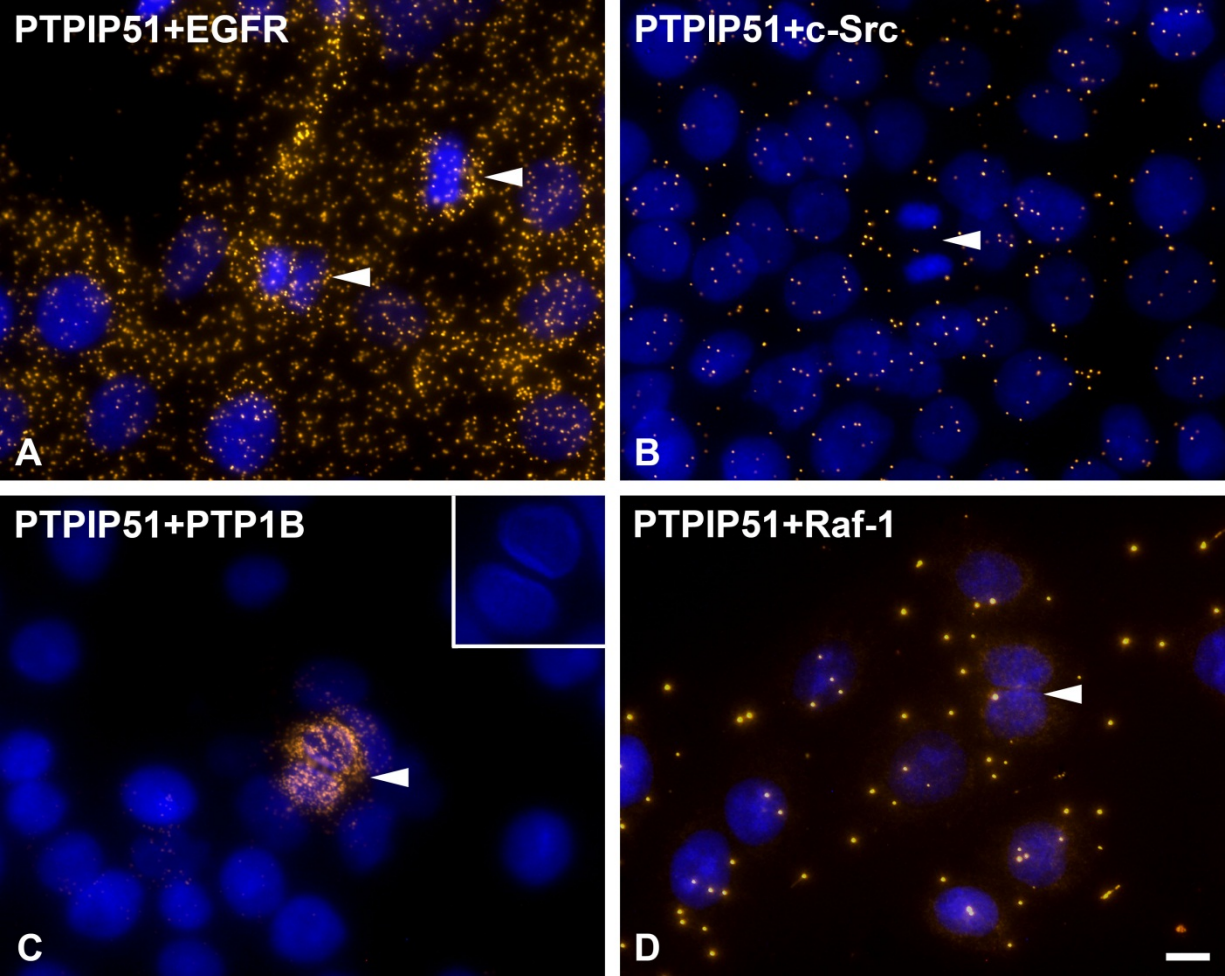
DPLA of the interaction partners of PTPIP51: EGFR, c-Src, PTP1B and Raf-1. (**A**) DPLA of PTPIP51 and EGFR. Mitotic cells are marked by arrows. (**B**) DPLA of PTPIP51 and c-Src. Arrow: mitotic cell. (**C**) DPLA of PTPIP51 and PTP1B. Dividing cell is marked by the arrow. The inset shows the nuclei of the dividing cell marked by the arrow. (**D**) DPLA of PTPIP51 and Raf-1. Dividing cell is marked by the arrow. PTPIP51 antibody: P51ab. Nuclei are marked by Dapi. Bar: 10 µm.

In mitosis as well as in interphase PTPIP51 interacted with the intracellular part of the epidermal growth factor receptor (EGFR), with c-Src, with Raf-1 and with PTP1B as seen by Duolink proximity ligation assay (Figure 10). The magnitude of these interactions strongly varied during the cell cycle (Figure 10). The interaction of PTPIP51 with EGFR was highest in mitotic cells (arrows) compared to interphase cells as seen in Figure 10A. In contrast, PTPIP51 and c-Src interaction was lowest in mitotic cells (Figure 10B, arrow). In interphase cells the number of DPLA spots indicating the PTPIP51 and c-Src interaction were measured in higher amounts (Figure 10B). Such a cell cycle dependent interaction pattern also was seen in cells tested for PTPIP51 and PTP1B interaction. PTPIP51 and PTP1B interaction was highest in post-mitotic cells undergoing cell division (Figure 10C, arrows). The inset in Figure 10C shows nuclei of dividing cells. Moreover, PTPIP51 and Raf-1 interaction levels also varied during cell cycle progression (Figure 10D). Highest interaction levels were found in interphase cells (Figure 10D), whereas mitotic cells displayed single or no DPLA spots for PTPIP51 and Raf-1 interaction (Figure 10D, arrow).

## 4. Discussion

Keratinocytes grown under normal conditions were submitted to cell cycle synchronization by nocodazole. The synchronized cells were grown under standardized conditions. After restart of the cell cycle, cells were harvested at defined time points. PTPIP51 protein was highest 2 hours after the restart of the cell cycle where mitotic cells were most numerous (data not shown), whereas the mRNA expression reached a maximum 6 hours post synchronization.

Synchronized HaCat cells expressed different molecular forms of unphosphorlyated-PTPIP51 and phosphorylated-PTPIP51. The exact function of the phosphorylation at tyrosine residue 176 of PTPIP51 is still unknown. The absence of the 60 kDa phosphorylated PTPIP51 form 4 hours post synchronization probably hints to an involvement in mitotic processes. Two studies from our laboratory pointed to a regulation of the PTPIP51/Raf-1 interaction by tyrosine phosphorylation [8,9]. In cells of acute myeloid leukemia PTPIP51 is unable to interact with Raf-1 while phosphorylated at its tyrosine 176 residue [9]. In general, this indicates a mechanism for conducting PTPIP51 to specific cellular compartments and specialized cellular functions. This is corroborated by the present data. In mitotic cells PTPIP51 is almost completely phosphorylated at its tyrosine 176 residue. In interphase cells tyrosine 176-phosphorylated PTPIP51 is found in a more dispersed manner. Oishi and co-workers [11] found ectopically expressed PTPIP51 in association with the spindle pole of the forming spindle apparatus in mitotic cells. Such a co-localization is substantiated by the present study. In addition, the co-localization of the tyrosine-phosphorylated form with tubulin at the spindle pole was observed. Moreover, the direct interaction with tubulin was corroborated. These facts point to a pivotal role of tyrosine phosphorylated PTPIP51 in the formation of the spindle apparatus. The spindle apparatus enucleates from the doubled centrosome. Here, ninein is a key player for correct enucleation and anchorage of the minus ends of the microtubules [30]. Ninein was found to interact with CGI-99 and thereby the phosphorylation of ninein by glycogen synthase kinase 3 β (GSK3β) is inhibited [15]. Interestingly, PTPIP51, CGI-99, ninein and GSK3β are located at the centrosome [15,31]. The complex of these four proteins probably regulates the polymerization of the microtubules. The PTPIP51/CGI-99 complex possibly influences the interaction of GSK3β and ninein and subsequently the association of ninein to the centrosome. Thus, ninein is unable to influence anchorage and enucleation of the microtubules. Yet, more data are needed to elucidate the full regulatory network formed by PTPIP51, CGI-99, ninein and GSK3β. Furthermore, ninein is released from the centrosome in speckles with restriction to the microtubular cytoskeleton. Ninein can be transported to and from the centrosome and may play a role in cell polarization of epithelial cells [31]. In this study PTPIP51 was associated with tubulin and CGI-99 in interphase cells. The interaction of PTPIP51 and CGI-99 could be traced throughout the whole cytoplasm. Thus, the interaction of PTPIP51/CGI-99 may also influence microtubular association and cell polarity by the modulation of ninein.

Furthermore, PTPIP51 interacts with the kinetochore protein Nuf-2 mediating correct recruitment of the microtubules to the kinetochore during mitosis [13]. Nuf-2 is part of the Ndc80 complex next to Hec1, Spc24 and Spc25 [32]. Spc24 and Spc25 anchor the complex into the kinetochore. Using these two proteins as a base, the N-terminal domains of Nuf2 and Hec1 interact with the plus ends of the spindle microtubules [33,34]. All four members of the NDC80 complex are predicted to inherit coiled-coil domains. The NDC is divided into two subcomplexes: the Hec1-Nuf-2 complex and the Spc24-Spc25 complex. These two subcomplexes are stabilized by the coiled-coil domains [35]. PTPIP51 is also found to have a coiled-coil domain [1]. Thus, PTPIP51 may be a member of the outer kinetochore NDC80 complex modulating anchoring and function of the spindle apparatus microtubules. In concordance, PTPIP51/tubulin interaction was also observed at the dividing chromosomes. This goes along with the observations of Kittler *et al.* [36] who reported defect cell division after knockdown of PTPIP51 gene. Summarizing these facts, we postulate that PTPIP51 plays a pivotal role in the formation of the spindle apparatus with impact on the minus and plus ends of the microtubules by its interaction with CGI-99 and Nuf-2.

As discussed earlier, the recruitment of PTPIP51 to the spindle apparatus is probably induced by its tyrosine 176 phosphorylation through c-Src [2]. Moreover, Brobeil and co-workers showed that Lyn, a member of the Src family kinases, also interacts with PTPIP51 in cells of acute myeloid leukemia [9]. Strikingly, during mitosis cdc2 phosphorylates c-Src at specific serine and threonine residues. In parallel, tyrosine kinase activity is increased approximately two-fold and accessibility of its SH2 domain for binding relevant phosphotyrosine-containing ligands increases by about 15-fold [37]. In the present study cells undergoing mitosis showed reduced levels of PTPIP51/c-Src interaction. In contrast, PTPIP51/EGFR interaction was increased in mitotic cells mirroring the main phosphorylation event of PTPIP51 at its tyrosine 176 residue during mitosis. Dangi and Shapira [38] showed that the downstream pathways of the EGFR signaling are uncoupled through phosphorylation mediated by the cdc2 kinase. During mitosis transcription and translation have to be inhibited due to their high energy demand. Extracellular regulated kinase (Erk) is the downstream molecule of the EGFR signaling coupled by the Ras-Raf-MEK cascade [38]. Cdc2 uncouples this cascade and there is no Erk activation during mitosis [38]. Moreover, PTPIP51 is known to activate Erk on Raf-1 level by its interaction with 14-3-3 protein and formation of a PTPIP51/14-3-3/Raf-1 complex [3]. This interaction is reduced if PTPIP51 is tyrosine phosphorylated [8]. Thus, the phosphorylation of PTPIP51 at its tyrosine 176 residue by the EGFR receptor with subsequent inhibition of Raf-1 activation may be a further control mechanism for correct mitosis and inhibition of Erk activity. This is in concordance with the acquired data on PTPIP51/Raf-1 interaction in post-mitotic dividing cells, where no or only single interactions of PTPIP51 with Raf-1 were observed. Interestingly, in post-mitotic dividing cells PTPIP51/PTP1B interaction is strongly increased. PTPIP51/PTP1B interaction is thought to be an enzyme-substrate complex [2]. Thus, PTP1B dephosphorylates PTPIP51 at its tyrosine 176 residue to function as a modulator of the mitogen activated protein kinase (MAPK) pathway in interphase cells.

## 5. Conclusions

In conclusion, PTPIP51 is a newly recognized protein with functional implication in spindle apparatus formation in mitotic cells. The recruitment of PTPPI51 is mediated through distinct tyrosine phosphorylation at the tyrosine 176 residue mediated by the EGFR. Tyrosine 176 phosphorylated PTPIP51 also ensures the uncoupling of the MAPK pathway to assure the energy supply for mitosis.

## References

[1] Brobeil A., Bobrich M., Wimmer M. (2011). Protein tyrosine phosphatase interacting protein 51—A jack-of-all-trades protein. Cell Tissue Res..

[2] Stenzinger A., Schreiner D., Koch P., Hofer H.W., Wimmer M. (2009). Cell and molecular biology of the novel protein tyrosine-phosphatase-interacting protein 51. Int. Rev. Cell Mol. Biol..

[3] Yu C., Han W., Shi T., Lv B., He Q., Zhang Y., Li T., Zhang Y., Song Q., Wang L., Ma D. (2008). PTPIP51, a novel 14-3-3 binding protein, regulates cell morphology and motility via Raf-ERK pathwa. Cell Signal..

[4] Lv B.F., Yu C.F., Chen Y.Y., Lu Y., Guo J.H., Song Q.S., Ma D.L., Shi T.P., Wang L. (2006). Protein tyrosine phosphatase interacting protein 51 (PTPIP51) is a novel mitochondria protein with an N-terminal mitochondrial targeting sequence and induces apoptosis. Apoptosis.

[5] Yao Y., Dai W. (2012). Mitotic checkpoint control and chromatin remodeling. Front Biosci..

[6] Hayne C., Tzivion G., Luo Z. (2000). Raf-1/MEK/MAPK pathway is necessary for the G2/M transition induced by nocodazole. J. Biol. Chem..

[7] Rosner M.R. (2007). MAP kinase meets mitosis: A role for Raf Kinase Inhibitory Protein in spindle checkpoint regulation. Cell Div..

[8] Brobeil A., Bobrich M., Tag C., Wimmer M. (2011). PTPIP51 in protein interactions—Regulation and in situ interacting partners.

[9] Brobeil A., Bobrich M., Graf M., Kruchten A., Blau W., Rummel M., Oeschger S., Steger K., Wimmer M. (2011). PTPIP51 is phosphorylated by Lyn and c-Src kinases lacking dephosphorylation by PTP1B in acute myeloid leukemia. Leuk Res..

[10] Löwenberg B., van Putten W.L., Touw I.P., Delwel R., Santini V. (1993). Autonomous proliferation of leukemic cells *in vitro* as a determinant of prognosis in adult acute myeloid leukemia. N. Engl. J. Med..

[11] Oishi K., Okano H., Sawa H. (2007). RMD-1, a novel microtubule-associated protein, functions in chromosome segregation in Caenorhabditis elegans. J. Cell Biol..

[12] Magidson V., O’Connell C.B., Lončarek J., Paul R., Mogilner A., Khodjakov A. (2011). The spatial arrangement of chromosomes during prometaphase facilitates spindle assembly. Cell.

[13] DeLuca J.G., Moree B., Hickey J.M., Kilmartin J.V., Salmon E.D. (2002). hNuf2 inhibition blocks stable kinetochore-microtubule attachment and induces mitotic cell death in HeLa cells. J. Cell Biol..

[14] Delgehyr N., Sillibourne J., Bornens M. (2005). Microtubule nucleation and anchoring at the centrosome are independent processes linked by ninein function. J. Cell Sci..

[15] Howng S.L., Hsu H.C., Cheng T.S., Lee Y.L., Chang L.K., Lu P.J., Hong Y.R. (2004). A novel ninein-interaction protein, CGI-99, blocks ninein phosphorylation by GSK3beta and is highly expressed in brain tumors. FEBS Lett..

[16] Porsche A. (2001). Identifikation von Interaktionspartnern der T-Zell Proteintyrosin-Phosphatase Durch das Lex-A Two Hybrid System. Ph.D. Dissertation.

[17] Olmsted J.B. (1981). Affinity purification of antibodies from diazotized paper blots of heterogeneous protein samples. J. Biol. Chem..

[18] Hofer W Buerklen T (2001). Specificity tests of the PTPIP51 antibody.

[19] Hofer W Schreiner D. (2004). Specificity tests of the PTPIP51 antibody.

[20] Stenzinger A., Kajosch T., Tag C., Porsche A., Welte I., Hofer H.W., Steger K., Wimmer M. (2005). The novel protein PTPIP51 exhibits tissue- and cell-specific expression. Histochem. Cell Biol..

[21] Barop J., Sauer H., Steger K., Wimmer M. (2009). Differentiation-dependent PTPIP51 expression in human skeletal muscle cell culture. J. Histochem. Cytochem..

[22] Koch P., Viard M., Stenzinger A., Brobeil A., Tag C., Steger K., Wimmer M. (2009). Expression profile of PTPIP51 in mouse brain. J. Comp. Neurol..

[23] Towbin H., Staehelin T., Gordon J. (1979). Electrophoretic transfer of proteins from polyacrylamide gels to nitrocellulose sheets: Procedure and some applications. Proc. Natl. Acad. Sci. USA.

[24] National Institutes of Health ImageJ. http://rsbweb.nih.gov/ij/index.html.

[25] Bob Dougherty Iterative Deconvolution. http://www.optinav.com/imagej.html.

[26] Bob Dougherty Diffraction Limit PSF. http://www.optinav.com/imagej.html.

[27] Li Q., Lau A., Morris T.J., Guo L., Fordyce C.B., Stanley E.F. (2004). A syntaxin 1, Galpha(o), and N-type calcium channel complex at a presynaptic nerve terminal: Analysis by quantitative immunocolocalizatio. J. Neurosci..

[28] University Health Network Research. Write Cell Imaging Facility. http://www.uhnres.utoronto.ca/facilities/wcif/fdownload.html.

[29] Gajadhar A., Guha A. (2010). A proximity ligation assay using transiently transfected, epitope-tagged proteins: Application for *in situ* detection of dimerized receptor tyrosine kinases. Biotechniques.

[30] Mogensen M.M., Malik A., Piel M., Bouckson-Castaing V., Bornens M. (2000). Microtubule minus-end anchorage at centrosomal and non-centrosomal sites: The role of ninein. J. Cell Sci..

[31] Moss D.K., Bellett G., Carter J.M., Liovic M., Keynton J., Prescott A.R., Lane E.B., Mogensen M.M. (2007). Ninein is released from the centrosome and moves bi-directionally along microtubules. J. Cell Sci..

[32] Sundin L.J., Guimaraes G.J., Deluca J.G. (2011). The NDC80 complex proteins Nuf2 and Hec1 make distinct contributions to kinetochore-microtubule attachment in mitosis. Mol. Biol. Cell.

[33] DeLuca J.G., Gall W.E., Ciferri C., Cimini D., Musacchio A., Salmon E.D. (2006). Kinetochore microtubule dynamics and attachment stability are regulated by Hec1. Cell.

[34] Wan X., O’Quinn R.P., Pierce H.L., Joglekar A.P., Gall W.E., DeLuca J.G., Carroll C.W., Liu S.T., Yen T.J., McEwen B.F., Stukenberg P.T., Desai A., Salmon E.D. (2009). Protein architecture of the human kinetochore microtubule attachment site. Cell.

[35] Ciferri C., de Luca J., Monzani S., Ferrari K.J., Ristic D., Wyman C., Stark H., Kilmartin J., Salmon E.D., Musacchio A. (2005). Architecture of the human ndc80-hec1 complex, a critical constituent of the outer kinetochore. J. Biol. Chem..

[36] Kittler R., Pelletier L., Heninger AK., Slabicki M., Theis M., Miroslaw L., Poser I., Lawo S., Grabner H., Kozak K., Wagner J., Surendranath V., Richter C., Bowen W., Jackson A.L., Habermann B., Hyman A.A., Buchholz F. (2007). Genome-scale RNAi profiling of cell division in human tissue culture cells. Nat. Cell Biol..

[37] Shalloway D., Bagrodia S., Chackalaparampil I., Shenoy S., Lin P.H., Taylor S.J. (1992). c-Src and mitosis. Ciba Found Symp..

[38] Dangi S., Shapiro P. (2005). Cdc2-mediated inhibition of epidermal growth factor activation of the extracellular signal-regulated kinase pathway during mitosis. J. Biol. Chem..

